# An integrated precision medicine approach in major depressive disorder: a study protocol to create a new algorithm for the prediction of treatment response

**DOI:** 10.3389/fpsyt.2023.1279688

**Published:** 2024-01-16

**Authors:** Bernhard T. Baune, Alessandra Minelli, Bernardo Carpiniello, Martina Contu, Jorge Domínguez Barragán, Chus Donlo, Ewa Ferensztajn-Rochowiak, Rosa Glaser, Britta Kelch, Paulina Kobelska, Grzegorz Kolasa, Dobrochna Kopeć, María Martínez de Lagrán Cabredo, Paolo Martini, Miguel-Angel Mayer, Valentina Menesello, Pasquale Paribello, Júlia Perera Bel, Giulia Perusi, Federica Pinna, Marco Pinna, Claudia Pisanu, Cesar Sierra, Inga Stonner, Viktor T. H. Wahner, Laura Xicota, Johannes C. S. Zang, Massimo Gennarelli, Mirko Manchia, Alessio Squassina, Marie-Claude Potier, Filip Rybakowski, Ferran Sanz, Mara Dierssen

**Affiliations:** ^1^Department of Mental Health, University of Münster, Münster, Germany; ^2^Florey Institute of Neuroscience and Mental Health, Parkville, VIC, Australia; ^3^Department of Psychiatry, University of Melbourne, Parkville, VIC, Australia; ^4^Department of Molecular and Translational Medicine, University of Brescia, Brescia, Italy; ^5^Genetics Unit, San Giovanni di Dio Fatebenefratelli Center (IRCCS), Brescia, Italy; ^6^Section of Psychiatry, Department of Medical Sciences and Public Health, University of Cagliari, Cagliari, Italy; ^7^Hospital del Mar Medical Research Institute (IMIM), Barcelona, Spain; ^8^Department of Adult Psychiatry, Poznan University of Medical Sciences, Poznan, Poland; ^9^Department of Mental Health, University Hospital Münster, Münster, Germany; ^10^Department of Science, Grants and International Cooperation, Poznan University of Medical Sciences, Poznan, Poland; ^11^Centre for Genomic Regulation (CRG), Barcelona, Spain; ^12^Department of Medicine and Life Sciences, Universitat Pompeu Fabra, Barcelona, Spain; ^13^Research Programme on Biomedical Informatics (GRIB), Hospital del Mar Research Institute (IMIM), Barcelona, Spain; ^14^Department of Mental Health and Addiction Services, ASST Spedali Civili of Brescia, Brescia, Italy; ^15^Section of Neuroscience and Clinical Pharmacology, Department of Biomedical Sciences, University of Cagliari, Cagliari, Italy; ^16^Gertrude H. Sergievsky Center, Columbia University Irving Medical Center, New York, NY, United States; ^17^Department of Pharmacology, Dalhousie University, Halifax, NS, Canada; ^18^Department of Psychiatry, Dalhousie University, Halifax, NS, Canada; ^19^Paris Brain Institute (ICM), National Centre for Scientific Research (CNRS), Paris, France

**Keywords:** major depressive disorder (MDD), treatment resistant depression (TRD), antidepressant treatment response, genomics, transcriptomics, predictive algorithm, patient empowerment, shared decision making (SDM)

## Abstract

Major depressive disorder (MDD) is the most common psychiatric disease worldwide with a huge socio-economic impact. Pharmacotherapy represents the most common option among the first-line treatment choice; however, only about one third of patients respond to the first trial and about 30% are classified as treatment-resistant depression (TRD). TRD is associated with specific clinical features and genetic/gene expression signatures. To date, single sets of markers have shown limited power in response prediction. Here we describe the methodology of the PROMPT project that aims at the development of a precision medicine algorithm that would help early detection of non-responder patients, who might be more prone to later develop TRD. To address this, the project will be organized in 2 phases. Phase 1 will involve 300 patients with MDD already recruited, comprising 150 TRD and 150 responders, considered as extremes phenotypes of response. A deep clinical stratification will be performed for all patients; moreover, a genomic, transcriptomic and miRNomic profiling will be conducted. The data generated will be exploited to develop an innovative algorithm integrating clinical, omics and sex-related data, in order to predict treatment response and TRD development. In phase 2, a new naturalistic cohort of 300 MDD patients will be recruited to assess, under real-world conditions, the capability of the algorithm to correctly predict the treatment outcomes. Moreover, in this phase we will investigate shared decision making (SDM) in the context of pharmacogenetic testing and evaluate various needs and perspectives of different stakeholders toward the use of predictive tools for MDD treatment to foster active participation and patients’ empowerment. This project represents a proof-of-concept study. The obtained results will provide information about the feasibility and usefulness of the proposed approach, with the perspective of designing future clinical trials in which algorithms could be tested as a predictive tool to drive decision making by clinicians, enabling a better prevention and management of MDD resistance.

## 1 Introduction

The World Health Organization (WHO) declared that “there is no health without mental health.” Mental health is a state of well-being in which an individual is aware of his or her own abilities, can cope with the normal stress of life, can work productively and is able to contribute to his or her community. Major depressive disorder (MDD) is the most common psychiatric disease worldwide and represents a leading cause of years lived with disability. In turn, this leads to an enormous socio-economic impact. Indeed, MDD represents the costliest psychiatric disorder in Europe (1). Moreover, it has been largely demonstrated that women are nearly twice as likely as men to be diagnosed with MDD. Different biological and environmental factors seem to increase the risk of depression in women; however, this issue remains largely unknown (2).

The main goal of treating MDD is to achieve remission and to maintain the therapeutic effects over time. Despite the availability of different classes of antidepressant drugs, the success of pharmacological treatment is still unsatisfactory, and matching a patient to his/her optimal treatment generally requires multiple trials of different treatments administered adequately in terms of doses and timing, with the sobering observation that the more treatments tried without success, the less likely a successful outcome. Only about 30 and 40% of patients experience remission after the first and second treatment course, respectively, and up to one third of them are classified as resistant to treatment (Treatment-Resistant Depression, TRD) (3, 4). This causes suffering for patients and their families and significantly contributes to pushing up costs for healthcare services.

The observation that TRD occurs despite the high variety of pharmacological drugs acting through different mechanisms of action suggests a possible common mechanism in resistant depression. This is consistent with evidence from studies that combine pharmacology, genetics, and brain imaging data, showing that non-response to a wide range of treatments share common etiology and common neuronal mechanisms that still need to be investigated (5).

Several clinical variables are associated with an unfavorable treatment outcome in MDD, such as earlier disease onset, greater severity, presence of psychiatric comorbidity, suicidal behaviors, and early life adversity (6). From a biological perspective, TRD is associated with specific molecular underpinnings, which are only partly known. Concerning transcriptomics, there is evidence of distinct patterns of gene expression, both in the central nervous system and in peripheral tissues, such as blood (7). Moreover, expression alterations of both coding genes and microRNAs (small non-coding RNAs that regulate gene expression) have been related to the lack of response to antidepressant treatment (8). In addition, several studies also indicated the existence of a genetic vulnerability to non-response to antidepressant drugs and TRD (7, 9). In animal models, RNA-seq on different brain regions after antidepressant treatments showed largely distinct gene changes associated with treatment response (10). Moreover, accumulating evidence shows that transcriptional changes seen across several brain regions in animal models of depression coincide with genetic risk factors in depressed human patients. This indicates the likelihood that peripheral changes in gene expression might reflect to some extent some aspects of brain function (11).

In this context, the identification of predictive markers will help the early detection of non-responder patients, who may be more prone to later develop TRD. However, the use of single sets of markers (either clinical or molecular) have shown limited predictive power and low replicability, indicating that the etiology of MDD in non-responder patients remains to be better understood. Through multi-omics integration, machine learning methods have the potential to model the interactions between several molecular layers (such as DNA or RNA) to predict a clinical endpoint using a holistic model (12). It is conceivable that the integration of diverse sets of predictors might increase the accuracy in the identification of non-responder and TRD patients.

The overall objective of the PROMPT (“Toward PrecisiOn Medicine for the Prediction of Treatment response in major depressive disorder through stratification of combined clinical and -omics signatures”) consortium, which is funded by the European ERA PerMed funding scheme, is to apply an integrated precision medicine approach in MDD through the combination of clinical, genomic, transcriptomic and sex-related data. The core objective is to create a new algorithm for the prediction of treatment response, which could be tested and validated in future clinical trials. This algorithm might represent a new tool for clinicians to drive decision-making, based not only on patients’ clinical features, but also on their genetic and transcriptomic background. An additional objective is to evaluate the potential use of a predictive pharmacogenetic tool in clinical practice from different perspectives and needs of various stakeholders involved in MDD treatment. Moreover, it is important to stress that the development of such an innovative precision medicine tool is central, but only part of the process to advance MDD treatment. Considering the later clinical application is crucial, and shared decision making (SDM) is increasingly viewed as the gold standard in patient-healthcare professional communication (13). SDM is a patient-centered approach that aids empowerment by supporting patients to actively take part in developing an informed decision about further treatment jointly with healthcare professionals based on clinical options as well as a patient’s individual preferences (14–16). Although SDM has been reported to lead to better decisions, increased patient participation, patient satisfaction, and treatment adherence and avoidance of overtreatment, its application in the mental health field is still rare (16, 17). Furthermore, multiple factors have been reported to influence SDM. This includes personal characteristics of the engaging parties, such as sex, age, clinical knowledge, years of experience, spoken language, or the level of education, factors relating to the interaction process, such as providing information or establishing a trustful relationship, and factors concerning broader structures of the healthcare system, for example, time constraints (18). In PROMPT, we consider application and SDM from the beginning and seek to identify factors that might come into play when patients and healthcare professionals come together to decide specifically about using the developed algorithm in clinical practice.

## 2 Methodology

### 2.1 Study design

**Figure 1:**
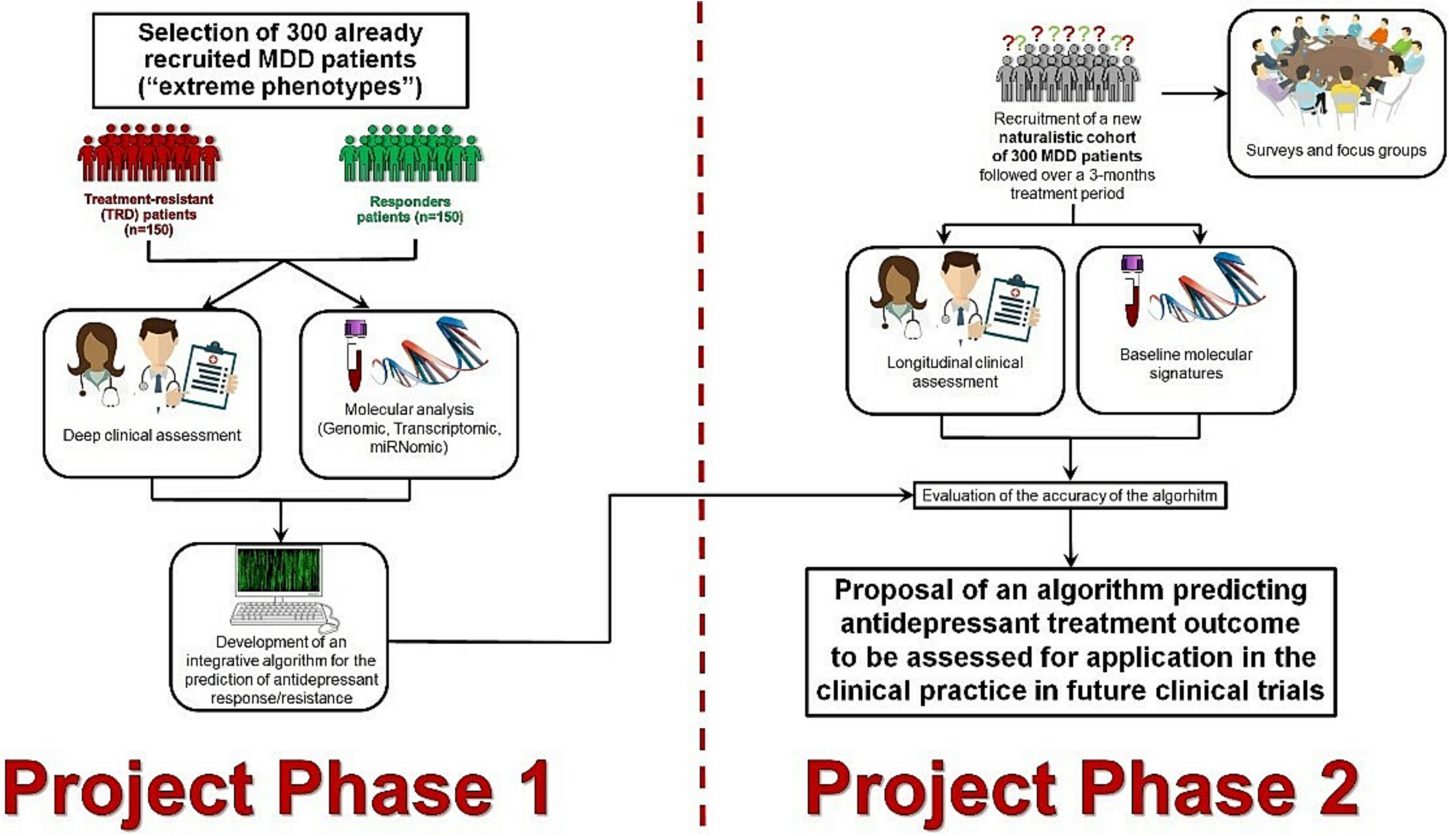
Study design phases of PROMPT project.

The overall methodology of the project is based on a two-phase design (Figure 1). In the first phase (training phase, retrospective design), 300 already recruited MDD patients, including 150 TRD and 150 responders considered as extremes phenotypes of response, will undergo a deep clinical and omics profiling. These data will be exploited to develop an innovative integrative algorithm for the prediction of MDD treatment outcome.

In the second phase (testing phase, prospective observational design), a new naturalistic cohort of 300 MDD patients will be recruited, and omics profiled to assess the predictive reliability of the algorithm under real-world conditions. Furthermore, in the second phase of the project, surveys involving the general population, patients as well as health care professionals, integrated with focus groups, will be performed on the topic of personalized, tool-assisted, and shared decision making processes. This will permit soundly to take into account the patients’ perspective, their needs on the use of predictive tools for MDD treatment and will support the process of patient empowerment in Personalized Psychiatry.

### 2.2 Phase 1: training phase

In this first project phase, two groups of clinically well-characterized MDD patients (TRD and responders), already recruited in the context of ongoing projects, will be selected considering them as extreme phenotypes of response allowing to train models on a dichotomous outcome. All patients will be profiled with genomic, pharmacogenetic, transcriptomic and miRNomic high-throughput technologies to create an integrative machine learning (ML) algorithm discriminating between the two groups.

#### 2.2.1 Study participants and clinical assessment

Three hundred MDD patients were already recruited from one unit participating in PROMPT consortium (IRCCS Fatebenefratelli, Brescia, Italy): half were classified as TRD and the other 150 as responders. For all of them, diagnosis of moderate to severe MDD according to the DSM-IV was confirmed using the Italian version of the SCID-I diagnostic scale. The diagnosis of personality disorders was made on the basis of clinical symptoms evaluation in agreement with the DSM-IV. Exclusion criteria were the following: (a) a lifetime history of schizophrenic, schizoaffective, or bipolar disorder; (b) personality disorder, substance abuse, alcohol abuse or dependency, obsessive compulsive disorder (OCD), or post-traumatic stress disorder (PTSD) as the primary diagnosis; (c) comorbidity with an eating disorders; (d) comorbidity with alcohol and substance dependence; (e) intellectual disability and cognitive impairment; (f) neurological disorders (i.e., Parkinson’s disease, multiple sclerosis, Alzheimer’s and other dementias, epilepsy, strokes, brain tumors, traumatic conditions of the nervous system); (g) comorbidity with other severe medical illness and severe autoimmune diseases (i.e., cancers, Crohn’s Disease, Rheumatoid Arthritis (RA), Lupus, Scleroderma, Psoriasis, Myasthenia gravis, Sjögren syndrome, Systemic lupus erythematosus); (h) pregnancy.

On the basis of clinical evaluation, TRD was defined as a failure of treatment to produce response or remission for patients after two or more treatment attempts of adequate and recommended dose and duration. Based on clinical judgment by the treating psychiatrists, MDD patients were classified as responders when they achieved response or remission in terms of a reduction in symptomatology with the first antidepressant treatment attempt of adequate dose and duration. For all patients, detailed socio-demographic (such as, age, sex, working and marital status) and clinical information (such as, age of onset, severity, psychiatric and physical comorbidities) was collected. Symptom evaluations were made using Montgomery-Åsberg Depression Rating Scale (MADRS) at the presentation of the patients to psychiatric services or hospital, in concomitance with the blood collection.

#### 2.2.2 Omics profiling

DNA and RNA extracted from peripheral blood samples are prepared for genomic, pharmacogenetic, transcriptomic and miRNomic profiling. DNA is extracted from whole blood samples using the Gentra Puregene Blood kit (Qiagen), according to the manufacturer’s instructions. Total RNA is extracted from blood already collected in PAXGene tubes and stored at −80°C with the PAXGene Blood miRNA Kit (Qiagen), designed for the simultaneous isolation of small and large RNAs. RNA is quantified and quality-checked through the Agilent 2,100 Bioanalyzer system and aliquots are sent to the involved project partners for transcriptomic and miRNomic profiling.

#### 2.2.3 Genomic and pharmacogenetic profiling

All the patients are genotyped through the GWAS array Infinium PsychArray-24 v1.3 BeadChip. In addition, all of them are genotyped with customized TaqMan OpenArray plates on a QuantStudio 12 K Flex Real-Time PCR System (Applied Biosystems, Foster City, California, United States) to obtain pharmacogenomics profile that include the following single-nucleotide polymorphisms (SNPs) in relative genes (15 in *CYP2D6*, 10 in *CYP2C19*, 4 in *CYP2B6*, 2 in *CYP2C9*, 8 in *CYP1A2*, 8 in *CYP3A4*, 11 in *ABCB1*). We also genotype the 5-HTTLPR (short/long allele) and rs25531 polymorphisms (A/G genotype) in the *SLC6A4* gene.

#### 2.2.4 Transcriptomic profiling

Abundant RNAs such as ribosomal and beta globin transcripts are removed starting from 10 ng total RNA using the Illumina Stranded total RNA Prep with Ribo-Zero Plus kit. RNA library preparation is performed following manufacturer’s recommendations. Final samples pooled library preparations are sequenced on a Novaseq 6,000 ILLUMINA, at a depth of 2x30Millions of 100bases reads per sample after demultiplexing (19).

#### 2.2.5 MiRNomic (+ other small RNA) profiling

MiRNomic (+ other small RNA) profiling is conducted by small RNA-Seq. The NEBNext^®^ Small RNA Library Prep Set for Illumina® kit is used with minor modifications. Adaptor ligation, first strand cDNA synthesis, and PCR enrichment are performed. Library amplification utilizes custom Unique Dual Indexes (UDIs). Purification steps involve AgenCourt AMPure XP beads, and library analysis is done using Agilent Bioanalyzer. Size selection is performed using 6% Novex TBE PAGE Gels, and quantification is carried out with the KAPA Library Quantification Kit. Sequencing yields 20–30 million single-end 50 bp reads per sample on a NextSeq2000 (Illumina).

#### 2.2.6 Bioinformatic analysis

Quality assessment is done with FastQC, and reads are trimmed using Cutadapt before mapping. For miRNOmic data, sequences with length < 16 nucleotides are discarded. Reads are aligned to the reference genome (hg38 and miRBase v22 for RNASeq and miRNOmic, respectively) with STAR. Counts table is generated using featureCounts, filtered for lowly expressed genes, and analyzed using linear models (limma) for differential expression analysis. Functional analysis utilizes available annotations in functional genomics resources. Network-based approaches are employed to visualize miRNA-target connections and perform gene ontology (GO) analyses. STRINGdb is used for protein–protein interaction retrieval, igraph for network analysis, and clusterProfiler for GO and pathway enrichment analyses. Differential expression of miRNAs is validated by qPCR.

#### 2.2.7 Sample size calculations

Power analyses were assessed using Bioconductor R packages ssizeRNA (20), ssize.fdr (21) and ssize (22). Parameters were obtained from seven publications of expression data in MDD patients (23–29). In cases where adjusted *p*-values were not reported, we adjusted them using the function p.adjust, with the FDR method. Dispersion of genes was not specified in the seven publications so we considered a 0.3 for all of them. Assessed experiments vary considerably in conditions, methods and results, which resulted in sample size estimations per group ranging between 11 and 121. Hence, we aim at a sample size of 150 per group, which exceeds the largest sample size calculated because we want to be conservative for the multi-omic nature of the study, but is also realistic considering our recruitment capacity.

#### 2.2.8 Integration of clinical and − omics data

With the purpose of understanding the molecular mechanisms of TR and identifying potential biomarkers to be used as features in a predictive model of treatment response (TR), we use multi-staged strategies such as differential gene/miRNA expression (limma), knowledge-driven miRNA-target analysis and Weighted Gene Co-expression Network Analysis (WGCNA), as explained previously. Nonetheless, given that we have three different omics layers (DNA, RNA miRNA), we also take advantage of meta-dimensional methodologies, which involve analyzing all omics layers simultaneously. These methodologies are especially powerful to capture complex interactions between the individual molecular layers and possibly identify new integrated molecular features (reduced dimensionality) that explain the phenotype. These new features are then being assessed, as features for a predictive model. We will employ different methods including iClusterPlus, which uses penalized likelihood approach with lasso penalty to associate a genomic feature with a phenotype, multi-omics factor analysis (MOFA), which infers an interpretable low-dimensional data representation as hidden factors or the partial least squares discriminant analysis (PLS-DA), implemented in mixOmics, which has increasingly been used in omics research as a supervised version of PCA that preserves in its first PC as much covariance as possible between the original data and its labeling (30). To avoid overfitting of the algorithm, this discovery analysis is done on two thirds of the Phase 1 data, keeping one third unseen from any training process.

Importantly, given the high relevance of the sex dimension in TR, we will stratify all analyses according to sex. This might as well help to further decipher the influence of sex on TRD. We also clinically assessed anxiety disorders in comorbidity, more frequently present in women, and will be analyzed with respect to omics data and putative sex effect.

#### 2.2.9 Development of the predictive algorithm

We will combine the multi-omic features identified to play a role in TR to generate a predictive model for TRD on Phase 1 data using state-of-the art statistical and machine learning methods for classification. We favor tree-based methods such as random forests or extreme gradient boosting over traditional regression models because they are not equipped to identify complex interacting risk structures empirically and have failed to model sex-specific associations (31). Standard methods of internal validation (e.g., bootstrap or cross-validation) will be used to estimate performance, to avoid over-fitting and to ensure reproducibility of the model. To select between models, we will use standard metrics such as Accuracy and F-measure on the validation set. Potential biases that may affect the inclusiveness of the models (e.g., sex or ethnicity issues) will be carefully considered.

### 2.3 Phase 2: testing phase

In the second phase of the project, the developed algorithm from phase 1 will be tested in a newly recruited naturalistic cohort of 300 patients to assess, under real-world conditions, the ability of the algorithm to correctly discriminate patients according to treatment response. Moreover, in the context of the new recruitment, patients’ focus groups and surveys will be set to assess perspectives and needs about predictive tools in precision medicine.

#### 2.3.1 Study participants and clinical assessment

A naturalistic cohort of 300 MDD patients is being recruited to assess, in real-world conditions, the capability of the algorithm to correctly predict the treatment outcomes. Patients are recruiting by the Department of Psychiatry at the University of Münster (Germany), by the Department of Medical Sciences and Public Health in Cagliari (Italy) and by the Department of Adult Psychiatry at Poznan (Poland). The broad inclusion criterion is a diagnosis of moderate to severe MDD and an age over 18 years. The exclusion criteria for Phase 2 are the following: (a) a lifetime history of schizophrenic, schizoaffective, or bipolar disorder; (b) personality disorder, drug abuse disorder, alcohol misuse and abuse disorder, OCD, PTSD as primary diagnosis; (c) comorbidity with alcohol and substance dependence; (d) severe neurological disorders (e.g., multiple sclerosis, Parkinson, dementia; intellectual disability; debilitating medical disorders). Diagnoses are confirmed according to the DSM-5 using the SCID-5-CV (clinical version) and the SCID-5-PD (personality disorders) diagnostic scale. At the baseline (T0), the Childhood Trauma Questionnaire (CTQ) is administered.

Patients are treated with antidepressant (AD) in monotherapy or with complex psychopharmacology such as two ADs or AD associated with other drugs (second-generation antipsychotics, mood stabilizers, lithium, FT3/FT4). Combination with diverse types of ongoing psychotherapy is accepted, if initiated prior to baseline.

Clinical assessment will be performed at 5 time points: baseline (T0), 2 (T1), 4 (T2), 8 (T3), and 12 (T4) weeks, using the MADRS, the Beck Depression Inventory II (BDI-II), the Beck Anxiety Inventory (BAI), the Columbia-Suicide Severity Rating Scale (C-SSRS) and the UKU Side Effects Rating Scale. At all time-points except the T1, the Functioning Assessment Short Test 24 items (FAST), the Quality of Life Questionnaire (SF-36) and the Perceived Stress Scale-10 (PSS-10) are administered. Moreover, at T0 and at T3 the Repeatable Battery for the Assessment of Neuropsychological Status (RBANS) are applied for the evaluation of cognitive symptoms in MDD patients.

This study involving human participants was reviewed and approved by the Ethics Committee “Ethik-Kommission Westfalen-Lippe” (Münster, Germany, registration number: 2021-103-f-S). Based on the German ethics approval, local ethics approval was obtained at the other clinical trial sites. The patients/participants provided their written informed consent to participate in this study. The study protocol was registered on ClinicalTrials.gov as NCT05537558.

#### 2.3.2 Biospecimens

Fasting blood samples are collected at T0, T2, T3, and T4 in each clinical recruitment center, which perform the first pre-processing steps. One EDTA Tube for DNA extraction and PAXGene Blood RNA Tube collected at T0 are sent to the same unit (IRCCS Fatebenefratelli, Brescia, Italy) that performed the DNA and RNA extractions for phase 1 to have uniform laboratory standards and reduce biases using the same methods described above in phase 1. The omics profiling (genomic, pharmacogenetic, transcriptomic, miRNomic) are carried out in the same sites and with the same methods described in phase 1. All remaining samples of unused biospecimens [EDTA tube, PAXGene Blood RNA, plasma, serum collected at each time point as well as peripheral blood mononuclear cell (PBMC)] (collected at T0 and T3) are stored locally at the recruitment sites and at the end of the project will be sent to Coordinator site in Münster, where the PROMPT Consortium biobank will be established using Centraxx standards.

#### 2.3.3 Outcomes

Our study has three major outcomes. The primary outcome is symptom improvement at week 8, as measured by the percent change in the MADRS score from baseline. Secondary outcomes include response and remission rates at 4, 8, and 12 weeks according to the MADRS. Tertiary outcomes include: (1) changes in scores of self-reported depressive symptoms at 2, 4, 8, and 12 weeks compared with baseline, as measured by the BDI; (2) response and remission rate at 4, 8, and 12 weeks according to BDI-II; (3) changes in scores of anxiety symptoms at 2, 4, 8, and 12 weeks compared with baseline, as measured by the BAI; (4) changes in scores of suicidal risk at 2, 4, 8 and 12 weeks compared with baseline, as measured by the C-SSRS; (5) changes in scores of perceived stress at 4, 8 and 12 weeks compared with baseline, as measured by the PSS-10; (6) changes in scores of psychosocial functioning at 4, 8 and 12 weeks compared with baseline, as measured by the FAST and Quality of Life Questionnaire (SF-36); (7) changes in scores of cognitive symptoms at 8 weeks compared with baseline, as measured by the RBANS; (8) and side effects at 4, 8, and 12 weeks, as assessed by the UKU Side Effect Rating Scale.

The response is defined as a ≥ 50% decrease in the assessment of interest (MADRS, BDI-II) at weeks 4, 8, and 12 compared with the baseline. Remission is defined as a score of ≤9 for MADRS and ≤ 9 for BDI-II. Moreover, the response to treatment is also computed at each time point considering different thresholds of symptom reduction (>20, >50, and > 80%) on the MADRS total score, as well as on the BDI-II total score. This approach allows defining fast responders (>20% after 2 weeks), partial responders (>50%) and full responders (>80%) after 8 weeks as compared to non-responders (<50% change in MADRS score) at week 8.

#### 2.3.4 Sample size calculations

Considering an Area Under Receiver Operating Characteristic (AUROC) Curve of 0.8, given a proportion of 0.3 of TRD, a confidence interval width of 0.125 at 0.95 confidence level, we computed that a sample size of at least 272 MDD patients will be enough to validate the predictive algorithm developed in the phase 1 of the PROMPT project.

#### 2.3.5 Data management

The data management process is the responsibility of the project coordinator. Clinical and biological data collection, analysis, storage, security, and sharing are consistent with the standard operating procedures that ensure patient pseudonymization.

Several data sets are generated, stored and shared during the project, including clinical data and omics data (genomic, transcriptomic, miRNomic, methylomic, and metabolomic).

We use data and metadata standard for file names and directories, clinical data and omics data. Access to data is restricted to qualified members of the project team. During the project, each data set is locally stored (secure servers, controlled access and backup copies). Secure protocols for data transfer such as sftp in concordance with national and European GDPR regulations are being used. For after the project, raw omics data and associated clinical metadata will be anonymized and hosted at the European Genome-Phenome Archive (EGA), following the MINSEQE standards. Codes and scripts will be deposited in software repositories (e.g., GitHub).

#### 2.3.6 Data integration and testing of the algorithm and predictive accuracy

Phase 2 data will be used to externally validate the model. This new naturalistic cohort will be different in the nature of patients as well as their provenance. We will assess the performance of the model using different measures such as C-index, accuracy, true positive rate and false positive rate. We will compute these measures for the whole cohort as well as in stratified groups by sex, ethnicity, and country of origin to assess potential biases of the model.

We also want to address the challenge of designing algorithms and tools that are both usable and effective, which are the two main obstacles in the clinical application of advanced statistical and ML models based on multi-omics data. Interpretability, intended as the ability to appropriately explain the reasoning behind the predictions, will be considered as a mandatory component of the model and can be achieved by using intrinsically interpretable models like random forests, by evaluating the model structure and importantly the feature importance, for instance through model agnostic techniques such as SHAP (SHapley Additive exPlanations) (32).

### 2.4 Perspectives and perceptions about predictive testing in the treatment of depression

This part of the PROMPT project seeks to identify perspectives and perceptions that may play a role when patients and professionals engage in a shared decision making (SDM) process on the question whether to apply an algorithm to aid decision making on the use of antidepressants. SDM requires engagement of health care professionals and facilitates patient empowerment by taking a patient’s wishes, values, beliefs, attitudes and perspectives into account (14–16). We approach this question on the possible value of an algorithm in treatment settings by employing two methodological approaches, qualitative focus groups and quantitative (online) surveys. Taken together, these two approaches will allow us to learn about the perspectives of different stakeholders participating in MDD treatment toward the assumed use of a treatment decision-aiding algorithm. It is anticipated that these results have the potential to foster translation into clinical practice, especially shared decision making processes.

#### 2.4.1 MDD patients focus group

Employing an experience-driven bottom-up approach, patient focus groups will be conducted at all patient-recruiting PROMPT sites (Munster − Germany, Cagliari − Italy, Poznan − Poland) to learn about the perspectives of MDD patients toward the algorithm in a rather hypothesis-freeway (33, 34). Using a pre-developed protocol, MDD patients meeting the criteria for participation in the PROMPT phase 2 will be invited to take part in a 90 min group discussion together with 3–4 fellow patients of different sex, age, and MDD history. Trained moderators will lead through the three-step procedure. After a short introduction to share previous experiences with depression treatment, participating patients learn about the algorithm and are encouraged to freely voice and discuss their thoughts, concerns, hopes and perspectives before the session concludes with an overall summery. Details about the algorithm are provided by means of a graphical representation and moderators are instructed to seek a broad exploration of the issues raised by the participants and to employ a series of follow up questions targeting specific areas of potential relevance. All focus groups will be audio recorded. Patient anonymity is maintained by choosing pseudonyms during the discussion and by removing private information from the subsequently generated transcripts. Following transcription of all audio recordings, anonymized transcripts will be further translated into English. Qualitative content analysis is conducted upon both, native language transcripts and English translations using MAXQDA^®^. Drawing on a transcript-based classification scheme, two different coders will analyze patients’ statements, focusing particularly on hopes or concerns associated with the algorithm, as well as on issues related to the decision-making process when deciding for or against the application of the decision-aiding algorithm that is being developed in the PROMPT project. To gain a broad understanding of the patient’s perspective on the algorithm and its application, we plan to conduct 4–5 focus groups at each site.

#### 2.4.2 Online surveys

Employing a theory-driven top-down approach, we will further develop surveys to learn about the perspectives of MDD patients, psychiatrists, neurologists, general practitioners, scientists, and the general population in a more hypothesis-driven way. These surveys contain items presented to any participant group as well as target group specific items. For example, all participants are asked to complete a hypothetical decision-making scenario. In this scenario, the algorithm is introduced and participants have to choose. In case patients are addressed in the survey, they are asked whether they would agree to undergo testing. In case health care professionals are addressed, they would be asked whether they would recommend the use of a testing tool for their patients with depression. Completing the surveys, all participants are further asked to rate perceived importance of a set of SDM related variables for this particular decision scenario and to fill in scales meant to operationalize participants’ attitudes, beliefs or perspectives about genetics more generally. All surveys will be provided in English and in the different native languages of the PROMPT-Consortium participating countries and distributed either as a link to a REDCap based online version or as a paper version at all PROMPT Sites (French, German, Italian, Polish and Spanish). Using the R statistical environment (35), we will run linear mixed effect models on a pre-processed and random forest imputed dataset (36). Analyses will be conducted for each participant group, as a whole and in sex-specific manner.

## 3 Summary and conclusions

Our project aims at the development of a clinically useful algorithm model that integrates clinical data (wide range of symptomatology assessment, treatment side effects, presence of childhood trauma) and -omics data (genomic, pharmacogenetic, transcriptomic and miRNomic profiling) for the prediction of treatment response in MDD patients. The study results are framed in the context of precision psychiatry and personalized psychiatry to enable the tailoring of the right therapeutic strategy for the right person at the right time. To account for sex-specific MDD outcomes, all analyses in the project will be stratified according to sex to better understand the sex dimension of treatment response both in relation to biological factors, sex-related lifestyle and environmental factors. Moreover, our project deepens the knowledge and experience of the shared decision making process when using predictive algorithms to aid decision making in Psychiatry. Both, predictive computational tools and shared decision making processes constitute key components of the Personalized Psychiatry concept. The definition of TRD that we used is the commonly accepted clinical definition of two or more failed pharmacological treatments. Unfortunately, the absence of a validated definition of TRD is a major limitation from the viewpoints of translational research, treatment development, as well as clinical and policy decision-making. Indeed, for example neurostimulation techniques and evidence-based psychotherapy are not considered in the definition of TRD, which is a limitation of this definition. TRD patients should include particularly the non-remitters and recurrent MDD patients having a high probability to have a poor prognosis of the disorder. The pathway toward more targeted treatments in psychiatry requires a more precise delineation of the phenotype being evaluated, and this represents an important goal for current and future research in psychiatry.

## Data availability statement

The original contributions presented in the study are included in the article/supplementary material, further inquiries can be directed to the corresponding author/s.

## Ethics statement

The studies involving humans were approved by Ethik-Kommission Westfalen-Lippe der Ärztekammer Westfalen-Lippe, Münster, Germany (registration number: 2021-103-f-S). Based on the German ethics approval, local ethics approval was obtained at the other clinical trial sites. The studies were conducted in accordance with the local legislation and institutional requirements. The participants provided their written informed consent to participate in this study.

## Author contributions

BB: Conceptualization, Funding acquisition, Methodology, Project administration, Resources, Supervision, Writing – original draft, Writing – review & editing. AM: Funding acquisition, Methodology, Project administration, Resources, Writing – original draft, Writing – review & editing. BC: Writing – review & editing, Funding acquisition, Supervision. MC: Investigation, Resources, Writing – review & editing. JDB: Writing – review & editing, Data curation, Formal analysis. CD: Data curation, Writing – review & editing, Project administration. EF-R: Project administration, Writing – review & editing, Investigation, Resources. RG: Investigation, Resources, Writing – review & editing. BK: Writing – review & editing, Data curation, Project administration. PK: Data curation, Project administration, Writing – review & editing. GK: Writing – review & editing, Investigation, Resources. DK: Investigation, Resources, Writing – review & editing. MML: Resources, Writing – review & editing, Data curation, Project administration. PM: Writing – review & editing, Formal analysis. M-AM: Formal analysis, Writing – review & editing. VM: Writing – review & editing, Investigation, Resources. PP: Investigation, Resources, Writing – review & editing. JPB: Resources, Writing – review & editing, Formal analysis, Writing – original draft. GP: Resources, Writing – review & editing, Investigation. FP: Writing – review & editing, Investigation, Resources. MP: Investigation, Resources, Writing – review & editing. CP: Investigation, Resources, Writing – review & editing. CS: Investigation, Resources, Writing – review & editing, Project administration. IS: Investigation, Resources, Writing – review & editing. VW: Investigation, Resources, Writing – review & editing. LX: Resources, Writing – review & editing, Formal analysis. JZ: Writing – review & editing, Formal analysis, Investigation, Resources, Writing – original draft. MG: Writing – review & editing, Conceptualization, Funding acquisition, Supervision. MM: Investigation, Project administration, Resources, Writing – review & editing. AS: Investigation, Resources, Writing – review & editing, Project administration. M-CP: Conceptualization, Funding acquisition, Writing – review & editing, Investigation, Resources, Writing – original draft. FR: Conceptualization, Funding acquisition, Supervision, Writing – review & editing. FS: Conceptualization, Funding acquisition, Writing – review & editing, Supervision. MD: Conceptualization, Funding acquisition, Investigation, Resources, Writing – original draft, Writing – review & editing.
